# Formation Mechanisms of Micro-Nano Structures on Steels by Strong-Field Femtosecond Laser Filament Processing

**DOI:** 10.3390/nano16010037

**Published:** 2025-12-25

**Authors:** Liansheng Zheng, Shuo Wang, Yingbo Cong, Chenxing Wang, Haowen Li, Hongyin Jiang, Helong Li, Hongwei Zang, Huailiang Xu

**Affiliations:** 1 State Key Laboratory of Integrated Optoelectronics, College of Electronic Science and Engineering, Jilin University, Changchun 130012, Chinawshuo23@mails.jlu.edu.cn (S.W.); chenxing25@mails.jlu.edu.cn (C.W.); lhw24@mails.jlu.edu.cn (H.L.);; 2 School of Mechanical and Aerospace Engineering, Jilin University, Changchun 130012, China; congyb@jlu.edu.cn; 3 Institute of Atomic and Molecular Physics, Jilin University, Changchun 130012, China; 4 School of Optoelectronic Engineering, Xidian University, Xi’an 710071, China

**Keywords:** femtosecond laser filamentation, laser fabrication, stainless steel, micro/nanostructures, preferential valley ablation, marangoni effect

## Abstract

Functional steel surfaces engineered through tailored micro-nano structures are increasingly vital for various applications such as high-performance aerospace components, energy conversion systems and defense equipment. Femtosecond laser filament processing is a recently proposed remote fabrication technique, showing the capability of fabricating micro-nano structures on irregular and large-area surfaces without the need of tight focusing. Nevertheless, the mechanisms underlying the formation of filament-induced structures remain not fully understood. Here we systematically investigate the formation mechanisms of filament-induced micro-nano structures on stainless steel surfaces by processing stainless steel in three manners: point, line, and area. We clarify the decisive role of the unique core–reservoir energy distribution of the filament in the formation of filament-induced micro-nano structures, and reveal that ablation, molten metal flow, and metal vapor condensation jointly drive the structure evolution through a dynamic interplay of competition and coupling, giving rise to the sequential morphological transitions of surface structures, from laser-induced periodic surface structures to ripple-like, crater-like, honeycomb-like, and ultimately taro-leaf-like structures. Our work not only clarifies the mechanisms of femtosecond laser filament processed morphological structures on steels but also provides insights onto intelligent manufacturing and design of advanced functional steel materials.

## 1. Introduction

Surface micro-nano structures fabricated by a variety of techniques have attracted considerable research interest owing to their critical roles in tailoring material surface properties. Ultrafast laser filament-based surface processing techniques have been increasingly employed and proven effective for remote, rapid fabrication of micro-nano structures on irregular and large-area surfaces of a variety of materials in recent years [1,2,3,4,5]. A laser filament is generated through the intricate interplay between Kerr self-focusing and plasma-induced defocusing effects that arise when a strong-field femtosecond (fs) laser beam propagates through a transparent medium including gases, liquids and solids [6,7,8,9]. Notably, fs laser filaments are capable of maintaining high peak intensities over extended distances, ranging from tens of centimeters to kilometers, without requiring tight focusing [10]. The transverse intensity distribution of a filament is highly non-uniform and is generally divided into two primary regions: the core region and the surrounding energy reservoir region. The core region is located at the filament center, which exhibits a higher energy density (typically 10^13^–10^14^ W/cm^2^) and serves as the main zone for nonlinear interaction and material processing. The energy reservoir region is characterized with a lower intensity but a broader spatial extent, and functions as a continuous energy supply for the core region [6]. The filament core is typically around 100 μm in diameter, whereas the surrounding energy reservoir extends over a considerably broader area [6]. These characteristics make filaments particularly advantageous for surface processing of large-scale or geometrically complex targets, eliminating the requirement for intricate 3D motion systems and enabling efficient, scalable, and cost-effective surface modification [3]. For example, it was demonstrated that femtosecond laser filaments can generate large-area honeycomb microstructures on silicon for use as molds in flexible pressure sensors [4], as well as on metals for creating functional surfaces such as superhydrophobicity, anti-reflection, and anti-icing [1,3,11]. More recently, it was shown that filament processing can achieve remarkable corrosion resistance on stainless steel surfaces with excellent physical and chemical durability [12].

However, although mechanistic insights into femtosecond laser filament processing have been reported, they are often fragmented and limited to specific materials, irradiation parameters, or individual structural features. In particular, a systematic understanding of the morphological transitions and formation pathways of filament-induced surface microstructures remains insufficient. Most existing studies have primarily focused on macroscopic performance indicators, such as wettability and reflectivity, while the underlying structure evolution mechanisms have received comparatively less attention. Moreover, significant differences in laser–matter interaction mechanisms exist across different processing dimensions, including point, line, and area fabrication, owing to their distinct energy distributions and thermal accumulation behaviors. A unified experimental investigation addressing these aspects simultaneously is still lacking.

This study employed stainless steel as a representative material to systematically investigate the formation and morphological transitions mechanisms of filament-induced surface microstructures. A series of controlled experiments were performed across three spatial dimensions (point, line, and area) by precisely varying the number and spatial distribution of incident filament pulses. The resulting surface morphologies were characterized using scanning electron microscopy (SEM) and laser scanning confocal microscopy (LSCM), revealing distinct structures including laser-induced periodic surface structures (LIPSS), micrometer-scale ripples, crater-like, honeycomb-like textures, and taro-leaf-like features. The results indicate that ablation, molten metal flow, and metal vapor condensation collectively drive structure formation, with their relative contributions depending on processing conditions. Furthermore, we demonstrated that both the filament core and energy reservoir play vital roles in morphological transitions, distinguishing this method from conventional tightly focused fs laser processing. Overall, this work reveals the underlying mechanisms of filament-induced morphological structures of steels through a combination of experimental observation and theoretical analysis, providing a solid basis for the rational design of high-performance functional metal surfaces.

## 2. Materials and Methods

**Figure 1:**
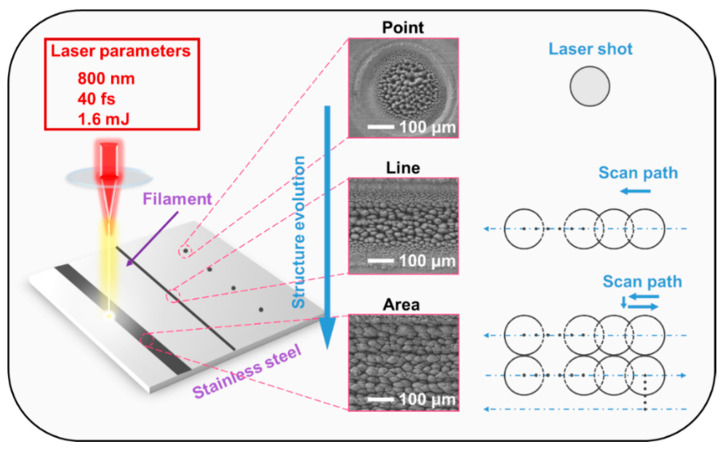
Schematic illustration of different types of femtosecond laser filament fabrications.

A Ti: sapphire femtosecond laser system (Spectra-Physics (Milpitas, CA, USA), Spitfire ACE) was employed to deliver linearly polarized laser pulses with a central wavelength of 800 nm, a pulse duration of 40 fs, and a repetition rate of 1000 Hz. The polarization direction of the incident linearly polarized laser was continuously adjusted by rotating a half-wave plate, thereby tuning the relative proportions of the p- and s-polarized components. The laser was then incident on a reflective polarizer at the Brewster angle, where the p-polarized component was transmitted while the s-polarized component was reflected. In the subsequent experiments, the reflected laser was used, corresponding to an s-polarized output. The pulse energy was adjusted to 1.6 mJ by rotating a half-wave plate in combination with a polarizer. The laser was focused by a fused silica lens with a focal length of 75 cm, forming a laser filament approximately 4 cm in length. A 304 stainless steel (Hefei Wenghe Metal Materials (Anhui, China), 06Cr19Ni10) sample was mounted on a motorized two-dimensional electric moving stage controlled by a computer and processed under three different fabrication modes: spot, line, and area. As illustrated in Figure 1, during the spot fabrication, the number of pulses (ranging from 10 to 1000) delivered to a fixed position on the sample surface was regulated by a mechanical shutter (DHC (Beijing, China), GCI-7103M) with the timing accuracy of 0.1 ms. In the line fabrication mode, the sample was moved unidirectionally while being irradiated with a defined number of pulses. For the area fabrication, raster scanning was performed across the sample surface, and the number of scan lines was gradually increased from a single line to full surface coverage by controlling the shutter. Surface structures were examined using scanning electron microscopy (SEM, JEOL (Tokyo, Japan), JSM-7500F) and laser scanning confocal microscopy (LSCM, Olympus (Tokyo, Japan), OLS4100), and for each experimental condition, the measurements were repeated three times at different locations to ensure reproducibility.

## 3. Results and Discussion

### 3.1. Spot Fabrication

**Figure 2:**
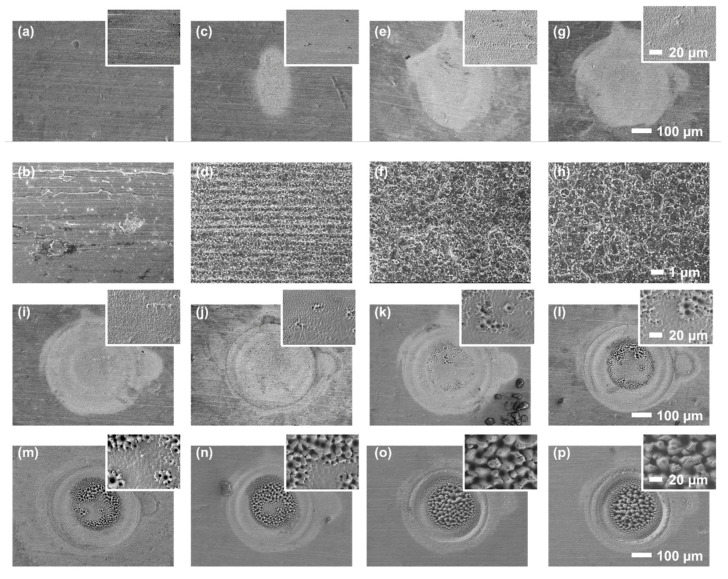
SEM images of sample surfaces ablated at a fixed point with different numbers of laser pulses: 15 (**a**,**b**), 16 (**c**,**d**), 17 (**e**,**f**), 20 (**g**,**h**), 27 (**i**), 40 (**j**), 70 (**k**), 100 (**l**), 140 (**m**), 200 (**n**), 500 (**o**), and 1000 (**p**). High-magnification SEM images are shown as insets.

To investigate the influence of pulse number on the formation and morphological transitions of surface micro-nano structures, a series of spot fabrication experiments were performed at fixed positions on the sample surface. Representative results from multiple trials are presented and discussed. As shown in Figure 2a,b, when only 15 laser pulses were applied, the stainless steel surface exhibited almost no detectable modification. Once the number of pulses increased to 16, the LIPSS began to appear with a spatial period of approximately 600 nm and an orientation perpendicular to the laser polarization direction (Figure 2c,d). The formation of LIPSS is generally attributed to the interference between the incident laser and surface scattered waves [13,14,15,16], or the excitation of surface plasmon polaritons [17,18], driven by light-matter interactions. At this stage, the surface modification is dominated by the formation of laser-induced periodic surface structures (LIPSS), which introduce an anisotropic surface response and can result in an elongated or elliptical modification footprint rather than a fully symmetric shape [19].

A further increase to 17 pulses resulted in a rapid structural transition. Ripple-like structures (3–5 μm in size), while the previously formed LIPSS were disrupted. The surface also displayed randomly distributed nanopits and nanoparticles (Figure 2f). This transformation is primarily governed by thermal effects. As the pulse number increased, the localized fluence rose accordingly, leading to deeper melting and higher surface temperatures, which in turn triggered phase explosion followed by rapid resolidification. These processes produced new ripple structures [20,21,22]. Moreover, under the influence of molten metal flow and cavitation bubble dynamics, the surface became covered with randomly arranged nanostructures, which were frequently observed superimposed on the subsequently developed microstructures [23,24,25].

With further irradiation, the ripples became progressively more well-defined (Figure 2g,h). When the pulse number reached 27, crater-like structures began to appear within the ripple valleys (Figure 2i). This phenomenon can be attributed to preferential energy deposition in the surface valleys, where incident light is more likely to be scattered, confined, and repeatedly reflected due to the local concave geometry. Such geometric confinement leads to localized fluence enhancement and more efficient thermal accumulation within the valleys, resulting in higher temperatures in the valleys than the surrounding ridges under identical irradiation conditions. As a consequence, material removal is initiated and subsequently proceeds more rapidly in the valley regions than on the protruding areas. This valley-selective material removal process is commonly referred to as preferential valley ablation (PVA) [26,27]. At the same time, the valleys, having absorbed more laser energy, attained higher temperatures, establishing a radial thermal gradient from the valley center to surrounding regions. This temperature gradient induces molten metal flow from the lower surface tension (hotter) valleys to the higher surface tension (cooler) crests via the Marangoni effect. These mechanisms collectively drove the transition from ripples to crater-like structures, which became increasingly deeper and wider with further pulse accumulation [28].

The morphological transition of crater-like structures exhibited a distinct pattern. First, the crater-like features show a grouped agglomeration, i.e., crater-like structures tend to appear in close proximity rather than being randomly distributed across the surface. This grouped agglomeration was facilitated by the combined effects PVA and Marangoni effects. Second, crater-like structures initially appeared at the periphery of the fabrication spot and then progressively propagated inward (Figure 2i–o). This expansion behavior was governed by the spatial energy distribution within the filament core, which is characterized by a centrally peaked fluence that gradually decreases toward the periphery, resulting in more intense melting at the center [29]. As a result, the central ripple structures became increasingly smoothed (insets in Figure 2i–k), thereby reducing the conditions favorable for crater formation, so that crater-like structures were more likely to form in the peripheral regions.

PVA plays an important role in the formation and evolution of both crater-like and honeycomb-like structures. In general, laser ablation leads to material removal from the surface. However, under the PVA condition, the ablation efficiency is significantly higher in concave regions than on protruding areas. Consequently, more material is removed from the valleys, leading to progressively deepened depressions, while the relative height of adjacent protrusions increases. When acting together with Marangoni-driven molten metal flow, which transports molten material from hotter valley regions toward cooler elevated areas, this asymmetric material redistribution amplifies the initial surface relief, producing deeper craters with higher rims and ultimately promoting the development and coalescence of crater-like and honeycomb-like structures.

As the number of pulses further increased, both the size and density of crater-like structures expanded. Eventually, adjacent craters coalesced, and new mounds formed along their outer edges. This iterative process gave rise to interconnected concave and convex features, forming honeycomb-like structures (Figure 2m–p). These honeycomb-like structures protruded above the original surface and were governed by both the Marangoni effect and the vapor-liquid-solid (VLS) growth mechanism [26]. Specifically, the uneven laser energy distribution caused by the complex surface morphology generated thermal gradients in the molten layer, driving upward molten metal flow via the Marangoni effect. Meanwhile, evaporated material from the valleys condensed on the structure tops, further promoting upward growth (VLS growth mechanism). This combined effect accounts for the increased height and hemispherical caps commonly observed at the tops of the honeycomb features.

### 3.2. Line Fabrication

Following the investigation of morphological transitions under the spot fabrication, line fabrication experiments were subsequently performed by translating the laser spot across the sample surface. Based on the previous spot fabrication results, the ablated region displayed a circular footprint with a diameter of approximately 200 μm (Figure 2). During the line fabrication, the scan speed was fixed at 0.5 mm/s and the laser repetition rate was maintained at 1000 Hz. In this process, the sample was moved unidirectionally, and the number of pulses delivered to the surface was accurately regulated by controlling the opening time of the shutter.

**Figure 3:**
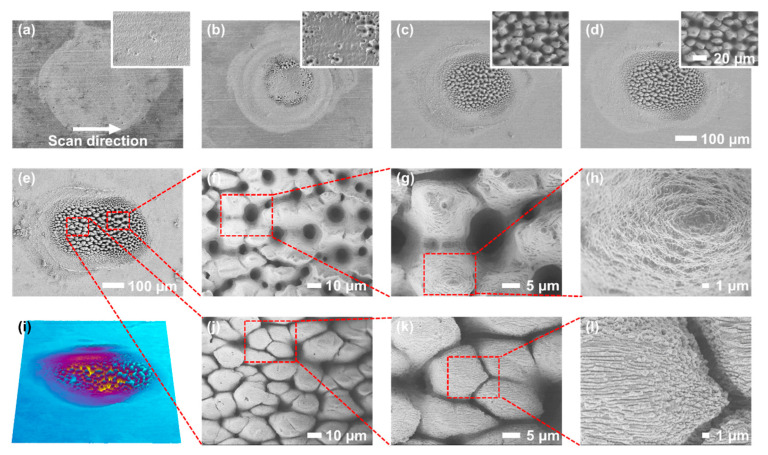
SEM images of sample surfaces fabricated in the line mode with different pulse numbers: 20 (**a**), 100 (**b**), 400 (**c**), 500 (**d**) and 1000 (**e**–**h**, **j**–**l**). High-magnification SEM images are shown as insets. (**i**) The 3D surface morphology of the sample ablated in line ablation mode with 1000 pulses. Different colours represent different surface heights.

At lower pulse numbers, the morphological transitions during the line fabrication followed trends similar to those observed in the spot fabrication case. As the pulse number increased from 20 to 200, the surface microstructures transitioned from initial ripple patterns to crater-like structures, and eventually to honeycomb-like formations. This transition, as in the spot fabrication case, also displayed an outward-to-inward propagation trend across the fabricated region (Figure 3a–c). However, distinct differences became evident at higher pulse numbers. As shown in Figure 3c–e, with a further increase in the pulse number, the continuous translation of the laser spot transformed the modified region from a circular footprint into an extended linear track. In the latter part of the scanning path, the surface retained honeycomb-like structures similar to those formed in the spot fabrication, characterized by rounded depressions and nanoscale wrinkles on the raised features (Figure 3e–h). In contrast, the front half of the scan track underwent a clear structural transition, particularly when the pulse number reached 1000 (Figure 3e,i). The original honeycomb-like structures transformed into a new type of morphology resembling taro-leaf-like structures (Figure 3j–l).

This structural transition is attributed to secondary melting effect induced by the energy reservoir region of the filament, which refers to the re-ablation and remelting of micro–nano structures initially formed by the filament core, caused by subsequent interaction with the surrounding energy reservoir region as the filament moves relative to the sample surface during scanning. During the filament formation, nonlinear effects such as Kerr self-focusing and plasma defocusing interact to produce a high-intensity clamped plasma channel at the center, referred to as the filament core, while the remaining laser energy spreads outward to form a lower-intensity but spatially broader energy reservoir region [6]. During scanning, the front segment of the scan path first interacted with the filament core region, leading to the formation of honeycomb-like structures. Subsequently, exposure to the surrounding energy reservoir region caused remelting and reconstruction of the original structures. This process resulted in the growth and fusion of adjacent raised structures (Figure 3k), thereby reducing the number of valleys and increasing the proportion of raised areas. Moreover, the structural peak shifted from being positioned above the original surface (as in honeycomb structures) to lying below it (Figure 3i), indicating that the formation of microstructures at this stage was primarily governed by ablation [26,30].

**Figure 4:**
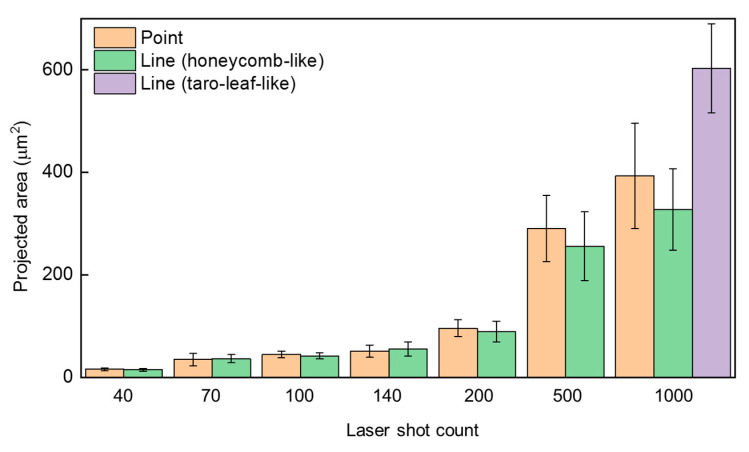
Projected area of micrometer-scale mounds versus the number of pulse irradiation with point and line fabrication. The projected areas are estimated by the SEM images shown in Figure 2 and Figure 3.

To quantitatively evaluate the morphological transition during filament processing, we measured the projected area of micrometer-scale mounds generated in both spot and line fabrication modes (Figure 4). In both cases, the projected area increased progressively with the number of laser pulses, even as the surface morphology transitioned from crater-like to honeycomb-like structures. This growth trend is primarily attributed to the combined influence of PVA and the Marangoni effect. At low pulse numbers (<200), the projected areas of structures formed in spot and line fabrication were approximately comparable. However, at higher pulse numbers, the honeycomb-like structures obtained from spot fabrication displayed significantly larger projected areas than those formed via line fabrication. This difference arises from the fact that, during the line fabrication, each point in the fabrication region was exposed to a limited number of pulses due to the continuous motion of the laser spot relative to the sample, whereas in the spot fabrication, laser energy accumulated repeatedly at the same location. At 500 pulses, the projected area of microstructures formed in the spot fabrication already exceeded that in the line fabrication, and this gap further widened at 1000 pulses. Notably, this difference was most pronounced during the honeycomb-like structure formation stage. Once taro-leaf-like structures emerged in the line fabrication process, the projected area of the resulting mounds approximately doubled compared to those of the honeycomb-like structures formed in the same mode, even surpassing those produced under the spot case. This behavior indicates that the projected-area comparison offers quantitative support for secondary melting effect, wherein the energy reservoir region of the filament further remelts and reconstructs the micro–nano structures initially formed by the filament core during the later stages of morphological transition.

### 3.3. Area Fabrication

**Figure 5:**
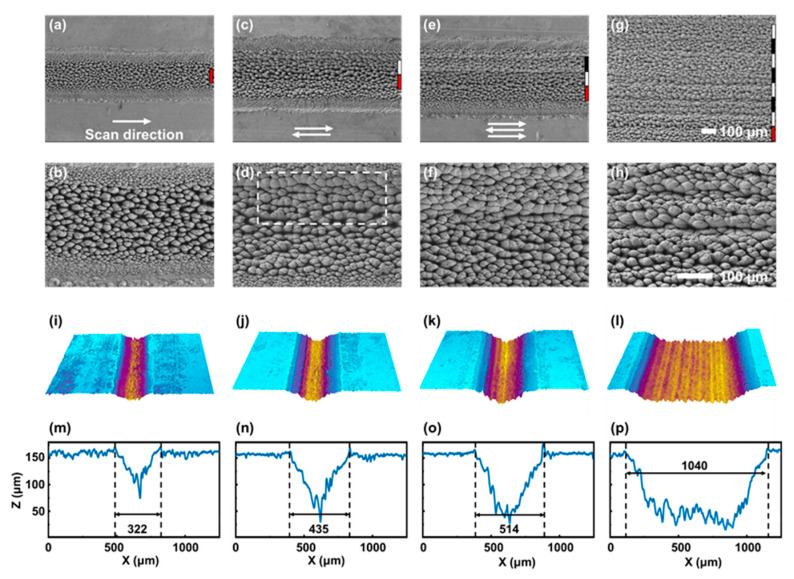
SEM images of sample surfaces after raster scanning with 1 (**a**,**b**), 2 (**c**,**d**), 3 (**e**,**f**), and 8 (**g**,**h**) scan lines. Each colored line segment on the side of the image corresponds to a length of 100 µm in the SEM images. 3D morphology and cross-sectional profiles of the sample surfaces after raster scanning with 1 (**i**,**m**), 2 (**j**,**n**), 3 (**k**,**o**), and 8 (**l**,**p**) scan lines. (For (**i**–**l**), different colours represent different surface heights. For (**m**–**p**), the regions marked by the dashed lines represent the widths of the grooves formed by laser fabrication).

When a raster scanning was performed over the sample surface for large-area modification, the resulting micro-nano structures exhibited characteristics distinct from those formed under the spot or line fabrication. As described in the previous section, the line fabrication typically produced taro-leaf-like structures (Figure 5a,b), because each surface location was sequentially irradiated by both the filament core and its surrounding energy reservoir as the beam translated. When two adjacent scan lines were separated by 100 μm, the microstructures in the region scanned first became noticeably larger than those produced by a single line pass (Figure 5c,d).

A direct comparison between the dashed white box in Figure 5d and the structures in Figure 5b illustrates this difference. Because the effective fabrication diameter of the filament core was approximately 190 μm, a 100 μm spacing corresponded to an overlap of about 95%, meaning that nearly the entire first track was re-irradiated during the second pass. This higher cumulative fluence further enhanced microstructure growth and led to significantly enlarged sizes. Similar behavior was observed when the number of scan passes increased to three and eight (Figure 5e–h). In each case, the final scan line generated smaller structures than those in previously irradiated regions, indicating that repeated laser exposure plays a crucial role in driving surface morphological transitions.

To quantify the cross-sectional profile and depth transition, the LSCM images of the fabricated structures were measured. As shown in Figure 5i–p, the microstructures created by the line fabrication generally lay beneath the initial surface [26]. With an increasing number of scans, the groove depth increased steadily, demonstrating that ablation dominates structure formation in both line and raster modes. Moreover, in Figure 5l, ridges composed of microscale mounds were periodically aligned parallel to the scanning direction, with a period of about 100 μm, indicating strong macroscale spatial regularity in filament-induced patterning.

**Figure 6:**
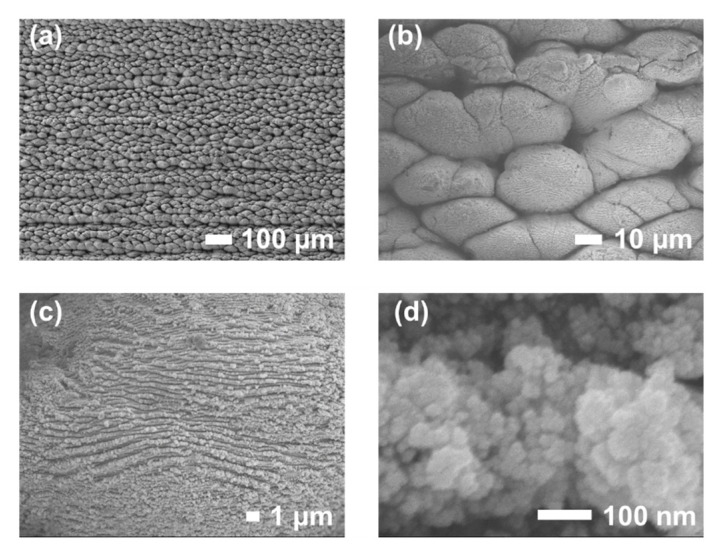
SEM images at different magnifications of the sample surface after raster scanning across the entire surface. SEM images at different magnifications of the sample surface after raster scanning across the entire surface, showing the first (**a**), second (**b**), third (**c**) and fourth (**d**) levels hierarchical structures, respectively.

After the raster scanning of the entire surface of the 304 stainless steel sample by the filament, the resulting surface morphologies were consistent with those observed in multi-line fabrication. The surface exhibited a typical four-level hierarchical architecture, described as follows:

(I) First-level structures: microscale ridges, with a spatial interval of approximately 100 μm, aligned parallel to the scanning direction and composed of microscale mounds (Figure 5h and Figure 6a);

(II) Second-level structure: individual mounds, typically measuring tens of micrometers in diameter (Figure 6b);

(III) Third-level structure: the surfaces of these mounds were densely covered with nanoridges or nanoprotuberances, typically several hundred nanometers in width (Figure 6c);

(IV) Fourth-level structure: at higher magnification, these nanostructures were revealed to consist of densely packed nanoparticles, each with a diameter on the order of tens of nanometers (Figure 6d).

**Figure 7:**
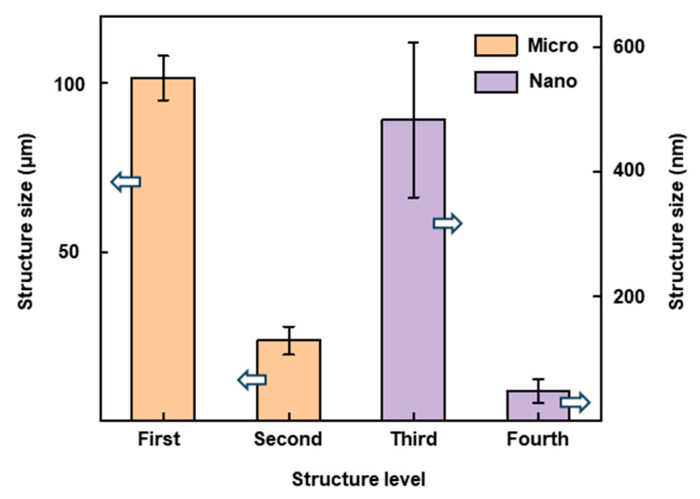
Quantitative size distributions of the four hierarchical structural levels induced by femtosecond laser filament processing. The arrow directions show the data represented by the corresponding y-axis.

Figure 7 presents the mean sizes and standard deviations of the four hierarchical structural levels, with the statistical data obtained from the five largest characteristic features identified in the SEM images in Figure 6. Notably, this hierarchical micro-nano structure was fabricated in a single-step laser processing procedure, without the need for multi-stage fabrication or post-treatment. After surface energy reduction to form a superhydrophobic state, the hierarchical micro–nano structures are favorable for long-term air-layer retention, which is crucial for maintaining durable superhydrophobic behavior [12,31]. These hierarchical features facilitate air-pocket retention and enhance capillary pressure at the solid–liquid interface, thereby preventing droplet impalement and improving the durability and long-term stability of the modified surface.

## 4. Conclusions

To summarize, the formation mechanisms of femtosecond laser filament-induced micro-nano structures on 304 stainless steel surfaces were systematically examined under three spatial processing modes: spot, line, and area. Comparative analysis across these dimensions reveals that the structure formation is governed by the combined action of ablation, molten metal flow, and metal vapor condensation, with the dominant process varying depending on the fabrication mode. A key finding is the pivotal role of the filament’s unique core-reservoir energy distribution, where the energy coupling between the high-intensity core region and the surrounding reservoir region substantially influences the morphological transition during scanning fabrication. In the spot fabrication case, as the number of pulses increased, the sample surface initially develops LIPSS, which are rapidly transformed into ripple-like structures. Subsequently, crater-like structures gradually form from the periphery toward the center of the ablation site, eventually transforming into honeycomb-like morphologies. In the line fabrication, the surface follows a similar morphological transition. However, due to the influence of the energy reservoir region, taro-leaf-like structures emerge when the number of pulses is sufficiently large. Ultimately, the area fabrication yields a four-level hierarchical micro-nano structure in a single processing step. Although the surface morphologies from ripple- to honeycomb-like structures have been reported previously [1], the present work has revealed a previously unobserved continuous evolution from ripple-, crater- to honeycomb-, and ultimately taro-leaf-like structures on steels, enabled by the unique core–reservoir energy-distributed femtosecond laser filament processing. These findings deepen the understanding of morphological transition mechanisms in filament-based processing.

## Data Availability

Data will be made available on request.
